# Reproductive Isolation Between Taxonomically Controversial Forms of the Gray Voles (*Microtus*, Rodentia; Arvicolinae): Cytological Mechanisms and Taxonomical Implications

**DOI:** 10.3389/fgene.2021.653837

**Published:** 2021-05-10

**Authors:** Tatiana I. Bikchurina, Fedor N. Golenishchev, Elena A. Kizilova, Ahmad Mahmoudi, Pavel M. Borodin

**Affiliations:** ^1^Laboratory of Recombination and Segregation Analysis, Institute of Cytology and Genetics, Siberian Branch of the Russian Academy of Sciences, Novosibirsk, Russia; ^2^Laboratory of Structural and Functional Genome Organization, Novosibirsk State University, Novosibirsk, Russia; ^3^Laboratory of Theriology, Zoological Institute, Russian Academy of Sciences, St. Petersburg, Russia; ^4^Department of Cytology and Genetics, Novosibirsk State University, Novosibirsk, Russia; ^5^Department of Biology, Faculty of Science, Urmia University, Urmia, Iran

**Keywords:** voles, meiotic abnormalities, hybrid sterility, reproductive isolation, taxonomic status

## Abstract

The formation of hybrid sterility is an important stage of speciation. The voles of the genus *Microtus*, which is the most speciose genus of rodents, provide a good model for studying the cytological mechanisms of hybrid sterility. The voles of the “*mystacinus*” group of the subgenus *Microtus* (2*n* = 54) comprising several recently diverged forms with unclear taxonomic status are especially interesting. To resolve the taxonomic status of *Microtus mystacinus* and *Microtus kermanensis*, we crossed both with *Microtus rossiaemeridionalis*, and *M. kermanensis* alone with *Microtus arvalis* “obscurus” and *M. transcaspicus* and examined the reproductive performance of their F1 hybrids. All interspecies male hybrids were sterile. Female *M. kermanensis* × *M. arvalis* and *M. kermanensis* × *M. transcaspicus* hybrids were sterile as well. Therefore, *M. mystacinus*, *M. kermanensis*, and *M. rossiaemeridionalis* could be considered valid species. To gain an insight into the cytological mechanisms of male hybrid sterility, we carried out a histological analysis of spermatogenesis and a cytological analysis of chromosome synapsis, recombination, and epigenetic chromatin modifications in the germ cells of the hybrids using immunolocalization of key meiotic proteins. The hybrids showed wide variation in the onset of spermatogenesis arrest stage, from mature (although abnormal) spermatozoa to spermatogonia only. Chromosome asynapsis was apparently the main cause of meiotic arrest. The degree of asynapsis varied widely across cells, individuals, and the crosses—from partial asynapsis of several small bivalents to complete asynapsis of all chromosomes. The asynapsis was accompanied by a delayed repair of DNA double-strand breaks marked by RAD51 antibodies and silencing of unpaired chromatin marked by γH2A.X antibodies. Overall, the severity of disturbances in spermatogenesis in general and in chromosome synapsis in particular increased in the hybrids with an increase in the phylogenetic distance between their parental species.

## Introduction

The concept of biological species lays stress on reproductive isolation as the main discriminant of species (Mayr, 1970; Coyne and Orr, 2004). Tests for reproductive isolation combined with detailed molecular genetic and cytogenetic analyses have been successfully applied to resolve taxonomical issues with the gray voles *Microtus* (Rodentia; Arvicolinae), one of the most speciose rodent genera. This integrated approach made it possible to establish the species status of two sibling forms *Microtus arvalis* and *Microtus rossiaemeridionalis* and to confirm the species status of some controversial forms, such as *M. ilaeus* and *M. transcaspicus* (Meyer et al., 1985, 1996; Malygin and Sablina, 1994; Meyer, 1994; Torgasheva and Borodin, 2016). In this study, we applied this approach to clarify the taxonomic status of another set of controversial forms of the subgenus *Microtus* and to gain an insight into the cytological mechanisms of hybrid sterility.

According to a recent revision (Shenbrot and Krasnov, 2005; Pavlinov and Lissovsky, 2012; Golenishchev et al., 2019), the subgenus *Microtus* comprises six nominal forms: *M. arvalis* (Pallas, 1778) (with two karyoforms, “arvalis” and “obscurus”), *M. rossiaemeridionalis* (Ognev, 1924), *M. ilaeus* (Thomas, 1912), *M. transcaspicus* (Satunin, 1905), *Microtus kermanensis* (Roguin, 1988), and *Microtus mystacinus* (De Filippi, 1865). The former two sibling species occupy vast areas in Eurasia, cohabitating in most of them. The distribution area of *M. transcaspicus* is limited to the Iranian and Turkmenian Kopet Dag and central Afghanistan (Mahmoudi et al., 2017). *M. ilaeus* is mosaically distributed in Uzbekistan, Kazakhstan, and Kyrgyzstan. *M. kermanensis* is known so far only from the high-altitude meadows of the Sarduiyeh plateau (Iran, prov. Kerman). *M. mystacinus* was found in Iran in several localities (West Elburz Mts. Zanjan, NE Elburz Mts. Golestan, Semnan, Central Elburz Mts. Tehran) (Mahmoudi et al., 2017).

**FIGURE 1:**
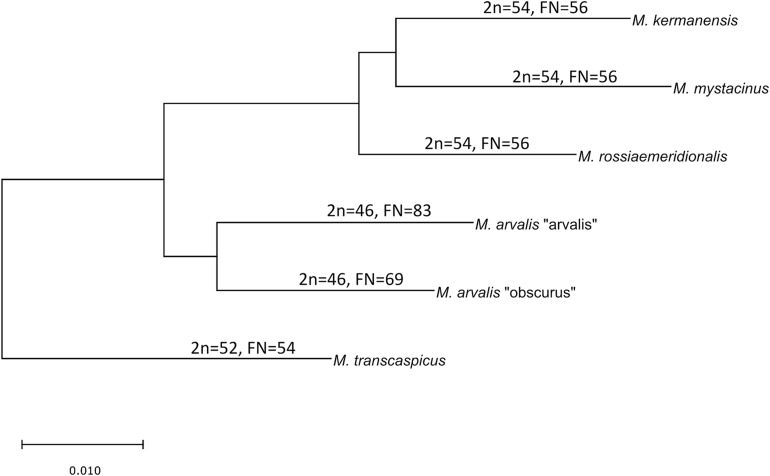
Consensus cladogram of the species of the subgenus *Microtus* based on Golenishchev et al. (2019).

The latter two species still have an unclear taxonomic status. *M. kermanensis* was originally thought to be a junior synonym for *M. transcaspicus* (Musser and Carlenton, 2005). However, the results of cytogenetic and morphological analyses and experimental hybridization indicated that *M. kermanensis* is closely related to *M. rossiaemeridionalis* (Golenishchev et al., 1999, 2000; Safronova et al., 2011). Until recently, *M. mystacinus* was considered a junior synonym for *M. arvalis* (Musser and Carlenton, 2005). However, Mahmoudi et al. (2014) suggested that, based on chromosome analysis, this form should be considered a valid name and showed its close affinity to the East European sibling vole *M. rossiaemeridionalis* (Mahmoudi et al., 2017). Later on, a comparative analysis of the *cytb* gene indicated that *M. kermanensis* and *M. mystacinus* form a clade separate from *M. rossiaemeridionalis* (Golenishchev et al., 2019). Figure 1 shows a consensus tree of the subgenus.

To clarify the taxonomic status of the “mystacinus” group (*M. mystacinus*, *M. kermanensis*, and *M. rossiaemeridionalis*) and their relation to the other species of the subgenus *Microtus* (*M. arvalis* and *M. transcaspicus*), which differ from each other both genetically and chromosomally, we examined the reproductive performance of their male and female hybrids. To detect disturbances in spermatogenesis, we carried out a conventional histological analysis. We used immunolocalization of key meiotic proteins to examine chromosome synapsis, recombination, and epigenetic chromatin modifications in the germ cells of the hybrids and to find the cytological basis of male sterility.

## Materials and Methods

### Animals

Voles trapped from natural populations were used as founders of short-term captive breed colonies maintained in the animal housing facilities of the Zoological Institute. In order to minimize the genetic heterogeneity of the colonies, we trapped a small number of the founders (four to five individuals) in a single population of each species over 1 week. The list of the trapping localities of the founders is shown in Table 1. The founders were used in intraspecies and interspecies crosses shown in Tables 2, 3. Breeding experiments lasted from 2017 to 2020. A total of 112 F1 hybrids in 34 litters were obtained (Table 2). The F1 hybrids were then backcrossed to the parental species (Table 3). The breeding pairs were kept together for up to 5 months. The pairs that did not produce progeny over this period were considered sterile. The age of the hybrid males used in the breeding experiments varied from 2.5 to 13 months. The ages of the males used for histological and cytological analyses are shown in Supplementary Table 1.

**TABLE 1:** List of the taxonomic groups examined.

Species	Locality	Latitude/longitude	2*n*	Reference for 2*n*
*M. kermanensis*	Iran, Kerman Province, Sarduiyeh *	29°14′9.60″/57°21′50.40″	54	Golenishchev et al., 2000
*M. mystacinus*	Iran, Mazandaran Province, Lasem*	35°47′50.97″/52°16′5.75″	54	Mahmoudi et al., 2018
*M. rossiaemeridionalis*	Russia, Leningrad District, Pushkin	59°48′1.76″/30°23′18.01″	54	Meyer et al., 1972
*M. arvalis* “arvalis”	Russia, Vladimir region	56°5′43.22″/40°54′14.06″	46	Matthey, 1952
*M. arvalis* “obscurus”	Russia, Sverdlovsk District	56°35′45.48″/61°06′0.58″	46	Orlov and Malygin, 1969
*M. transcaspicus*	Turkmenia, Kopetdag District, Firyuzin gorge*	37°54′0.00″/58°03′36.00″	52	Meyer and Orlov, 1969

*Vicinity of the type locality.

**TABLE 2:** Reproductive performance of the voles of the subgenus *Microtus* in interspecies crosses.

Dam	Sire	Abbreviations	*N* breeding pairs	*N* fertile pairs	Mean litter size
*M. kermanensis*	*M. kermanensis*	K	15	14	4.1 ± 0.3
*M. mystacinus*	*M. mystacinus*	M	15	13	3.2 ± 0.2
*M. rossiaemeridionalis*	*M. rossiaemeridionalis*	R	11	11	3.5 ± 0.3
*M. arvalis “arvalis”*	*M. arvalis “arvalis”*	Aa	11	10	3.6 ± 0.3
*M. arvalis “obscurus*	*M. arvalis “obscurus*	Ao	16	16	3.3 ± 0.3
*M. transcaspicus*	*M. transcaspicus*	T	5	4	2.7 ± 0.6
*M. kermanensis*	*M. mystacinus*	KM	2	2	4.0 ± 1.0
*M. mystacinus*	*M. kermanensis*	MK	1	1	4.0
*M. kermanensis*	*M. rossiaemeridionalis*	KR	3	3	3.7 ± 0.5
*M. rossiaemeridionalis*	*M. kermanensis*	RK	3	3	3.1 ± 1.0
*M. rossiaemeridionalis*	*M. mystacinus*	RM	3	3	2.7 ± 0.9
*M. mystacinus*	*M. rossiaemeridionalis*	MR	2	2	4.0 ± 0.0
*M. arvalis* “arvalis”	*M. kermanensis*	AaK	1	1	1.5 ± 0.5
*M. arvalis* “obscurus”	*M. kermanensis*	AK	3	2	4.0 ± 2.0
*M. kermanensis*	*M. transcaspicus*	KT	2	1	3.8 ± 1.5

**TABLE 3:** Reproductive performance of F1 interspecies hybrids of the subgenus *Microtus* in backcrosses to the parental species.

Dam	Sire	*N* breeding pairs	*N* fertile pairs	Mean litter size
*M. mystacinus*	F1 (*♀M. kermanensis* × *♂ M. mystacinus*)	2	0	
*M. rossiaemeridionalis*	F1 (*♀M. rossiaemeridionalis* × *♂ M. kermanensis*)	2	0	
*M. rossiaemeridionalis*	F1 (*♀M. rossiaemeridionalis* × *♂ M. mystacinus*)	2	0	
*M. mystacinus*	F1 (*♀M. rossiaemeridionalis* × *♂ M. mystacinus*)	2	0	
F1 (♀*M. kermanensis* × ♂ *M. mystacinus*)	*M. mystacinus*	2	1	1.0
F1 (♀*M. mystacinus* × ♂ *M. rossiaemeridionalis*) ×	*M. mystacinus*	2	2	2.8 ± 0.8
F1 (♀*M. rossiaemeridionalis* × ♂ *M. mystacinus*)	*M. rossiaemeridionalis*	2	2	4.0 ± 1.0
F1 (♀*M. kermanensis* × ♂ M. *rossiaemeridionalis*)	*M. kermanensis*	2	1	5.0
F1 (♀*M. rossiaemeridionalis* × ♂ *M. kermanensis*)	*M. kermanensis*	3	3	3.7 ± 1.6
F1 (*♀M. arvalis “obscurus*” × *♂ M. kermanensis*)	*M. kermanensis*	2	0	
F1 (*♀M. kermanensis* × *♂ M. transcaspicus*) ×	*M. kermanensis*	2	0	

*♀, dam; ♂, sire.*

The maintenance, handling, and euthanasia of animals were carried out in accordance with the national and international guidelines for the care and use of laboratory animals. All experiments were approved by the Ethics Committees for Animal Care and Use at the Zoological Institute and the Institute of Cytology and Genetics.

### Histological Analysis

The left testes of adult males were isolated immediately after euthanasia. Testicular tissues were separated from the *tunica albuginea* and fixed in 10% buffered formalin for 48 h. The samples were dehydrated in a graded ethanol series, immersed in xylene, and embedded in paraffin. Then, 7-μm-thick sections were cut using a sliding microtome and mounted on slides. The sections were deparaffinized, stained routinely with hematoxylin and eosin, and examined under an Axioskop 2 Plus microscope (Carl Zeiss, Jena, Germany) equipped with a CCD camera AxioCam HRc (Carl Zeiss) and AxioVision image-processing package (Carl Zeiss). The seminiferous epithelium cycle at the cross sections of the testes was described according to Leblond and Clermont (1952) and Wing and Christensen (1982). The cauda epididymis was minced in phosphate-buffered saline (PBS) at room temperature. Sperm morphology was examined at the smear prepared on glass slides. Histological evaluation was performed using a double-blinded technique on the samples and slides.

### Detection of Apoptotic Cells in Seminiferous Tubules Using TdT-Mediated dUTP Nick-End Labeling

The sections of each sample were deparaffinized, washed in PBS followed by fixation in 4% paraformaldehyde. Apoptosis was analyzed by a terminal deoxynucleotidyl transferase-mediated deoxyuridine triphosphate nick end labeling (TUNEL) assay using the DeadEnd Fluorometric TUNEL System (Promega, United States). Slides were mounted with Vectashield Antifade mounting medium (Vector Laboratories, Burlingame) to reduce fluorescence fading and examined under the Axioscop 2 plus microscope (Carl Zeiss, Germany) as described above.

### Synaptonemal Complex Spreading and Immunostaining

Chromosome spreads were prepared from the right testes of the males used in histological analysis. We used the drying down technique of Peters et al. (1997). Immunostaining was performed according to the protocol described by Anderson et al. (1999) using rabbit polyclonal anti-SYCP3 (1:500; Abcam), mouse monoclonal anti-SYCP3 (1:120; Abcam), mouse monoclonal anti-MLH1 (1:30; Abcam), rabbit polyclonal anti-γH2A.X (1:330; Abcam), rabbit polyclonal anti-SYCP1 (1:500; Abcam), rabbit polyclonal anti-RAD51 (1:250; Calbiochem), and human anticentromere (ACA) (1:70; Antibodies Inc.) primary antibodies. The secondary antibodies used were Cy3-conjugated goat anti-rabbit (concentrations varied from 1:500 to 1:100 depending on the primary antibodies used; Jackson ImmunoResearch), fluorescein isothiocyanate (FITC)-conjugated goat anti-mouse (concentration varied from 1:30 to 1:300 depending on primary antibodies used; Jackson ImmunoResearch), and aminomethylcoumarin (AMCA)-conjugated donkey anti-human (1:40; Jackson ImmunoResearch) antibodies. Antibodies were diluted in PBT (3% bovine serum albumin and 0.05% Tween 20 in PBS). A solution of 10% PBT was used for blocking. Primary antibody incubations were performed overnight in a humid chamber at 37°C; secondary antibody incubations lasted 1 h at 37°C. Slides were mounted with Vectashield antifade mounting medium (Vector Laboratories, Burlingame) to reduce fluorescence fading.

The preparations were visualized under the Axioplan 2 microscope (Carl Zeiss) equipped with a CCD camera (CV M300, JAI Corporation, Japan), CHROMA filter sets, and ISIS4 image-processing package (MetaSystems GmbH, Altlußheim, Germany). The images were preprocessed using the graphics editor Corel PaintShop Pro X6 (Corel Corporation, Canada).

The lengths of synaptonemal complexes (SCs) and their synapsed and asynapsed regions were measured using the MicroMeasure 3.3 program (Reeves, 2001). The number of recombination nodules per cell marked by MLH1 foci was scored in the pachytene cells, where all autosomal bivalents were completely synapsed and each bivalent contained at least one MLH1 focus.

### Data Analysis

Data in this paper are presented as mean values and standard deviations (mean ± SD). Pearson’s Chi-squared test was used to compare the observed and expected spermatocyte-to-spermatogonia and spermatid-to-spermatocyte ratios. Welch’s two-sample *t*-test was used to compare the total number of MLH1 signals per cell. All analyses were carried out in the R (v3.6.0) environment (R Core Team, 2019) for statistical computing using the packages BSDA (v1.2.0) (Arnholt and Evans, 2017), ggplot2 (v.1.0.7) (Wickham, 2016), and factoextra (v.3.2.1) (Kassambara and Mund, 2020).

To visualize histological data, we carried out a principal component analysis (PCA) for all specimens. While describing different groups of F1 hybrids, we considered six qualitative traits, such as gonadal morphology, seminiferous epithelium cycle, seminiferous tubule morphology, and the state of the seminiferous cell population (spermatocytes, spermatids, and spermatozoa) (Supplementary Materials 1, 2). These qualitative morphological features were scored on a grading scale of 0–3 (Supplementary Tables 2, 3) and thus converted into rank criteria. To make the qualitative description more accurate and user-friendly, we composed six block schemas (Supplementary Material 1 and Supplementary Figures 1–6). Together with these qualitative traits, we used three quantitative ratios: spermatogonia-to-Sertoli cells, spermatocytes-to-spermatogonia, and spermatids-to-spermatocytes (Supplementary Table 1). Next, we centered and normalized histological quantitative data on nine traits. Data on the (1) importance of components and (2) trait contribution to the PC are given in Supplementary Tables 4, 5. The algorithm of analysis we developed to represent histological data can be used in other studies of spermatogenesis.

## Results

### Production of the Hybrids

F1 hybrids were born in all variants of the interspecies crosses (Table 2). This indicates that there is no intrinsic pre-copulative isolation between any pair of the parental species, at least, in captivity. Litter size varied around 3.5, which is within the limits of variation characteristic of purebred species of this group (Table 2). Differences between crosses of different species and between intra- and interspecies crosses of the same species were not significant (Student’s *t*-test, *p* > 0.05), suggesting normal viability of the hybrid embryos.

### Reproductive Performance of the Hybrids

In order to estimate the fertility of the hybrids, we crossed them with the parental species. Table 3 shows that all hybrid males were completely sterile. None of them sired a progeny in any cross. Female hybrids between *M. kermanensis* and two distant species, *M. arvalis* “obscurus” and *M. transcaspicus*, were sterile. All F1 female hybrids from the *M. kermanensis* × *M. mystacinus* and *M. kermanensis* × *M. rossiaemeridionalis* and *M. mystacinus* × *M. rossiaemeridionalis* crosses were fertile.

Thus, the breeding data indicate that all the taxonomic groups examined can be considered valid species. They all show intrinsic postzygotic isolation in the form of hybrid sterility. Hybrid sterility is more advanced between the *M. kermanensis* × *M. arvalis* and *M. kermanensis* × *M. transcaspicus* hybrids, with both sexes being sterile, than between the *M. kermanensis* × *M. mystacinus* and *M. kermanensis* × *M. rossiaemeridionalis* and *M. mystacinus* × *M. rossiaemeridionalis* reciprocal hybrids, who show male sterility and female fertility.

### Dynamics of Spermatogenesis in the Interspecies Hybrids

The breeding test detected male hybrid sterility in all interspecies crosses. To gain an insight into the causes of this sterility and to estimate the degree to which spermatogenesis was affected, we carried out a histological examination of the testes of the hybrid voles.

**FIGURE 2:**
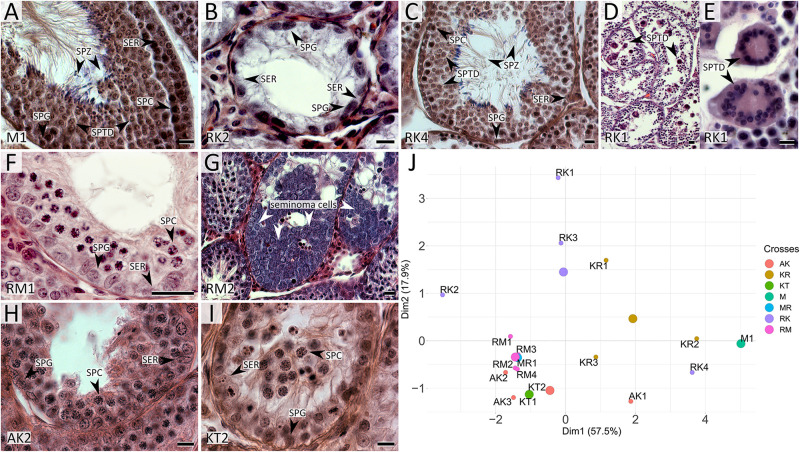
Histological sections of testes of *M*. *mystacinus* **(A)** and *M. rossiaemeridionalis* × *M. kermanensis* **(B–E)**, *M. rossiaemeridionalis* × *M. mystacinus* **(F,G)**, *M. arvalis* × *M. kermanensis* **(H)** and *M. kermanensis* × *M. transcaspicus* **(I)** hybrids stained by hematoxylin–eosin and PCA analysis of histological traits of F1 vole hybrids **(J)**. SER, Sertoli cell; SPG, spermatogonium; SPC, spermatocyte; SPTD, spermatid; SPZ, spermatozoon. Letters and numbers in the bottom-left corners of the graphs represent the type of cross and individual specimen number. Bar: 10 μm. In image J: small dots represent specimens, large dots represent centers of the clusters of one cross.

**FIGURE 3:**
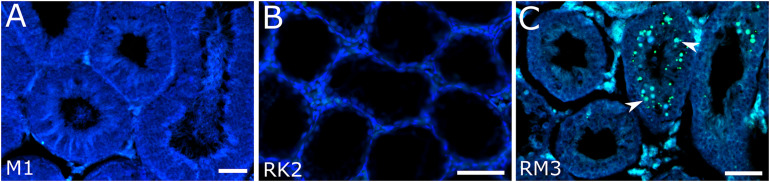
Detection of apoptotic cells in seminiferous tubules in M. *mystacinus* **(A)**, *M. rossiaemeridionalis* × *M. kermanensis* **(B)** and *M. rossiaemeridionalis* × *M. mystacinus* **(C)** hybrids using TdT-mediated dUTP nick-end labeling (TUNEL). The blue channel is DAPI, the green channel is TUNEL. Arrowheads point to apoptotic cells. Letters and numbers in the bottom left corners of the graphs represent the type of cross and individual specimen number. Bar: 50 μm.

We examined one *M. mystacinus* specimen as the control. Histological analysis revealed the normal structure of the testes. We detected no abnormalities in the seminiferous epithelium cell cycle (Figure 2A). The mean spermatid-to-spermatocyte I ratio was 3.4–1 and did not differ from the expected 4-to-1 ratio (χ^2^ = 0.03, *p* = 1.00) (Supplementary Table 1). The spermatocyte-to-spermatogonia ratio was 1.6–1, which did not differ from the expected 1-to-1 ratio (χ^2^ = 0.90, *p* = 0.97). TUNEL assay did not detect apoptotic cells in the tubules of this male (Figure 3A).

#### Hybrids Between *Microtus kermanensis* and *Microtus rossiaemeridionalis*

Seven hybrids obtained in both directions of reciprocal crosses showed a variety of disturbances in spermatogenesis: from its complete arrest in the very beginning to almost normal spermatogenesis proceeding to term and producing mature but mostly abnormal sperm.

RK2 had severally distorted testes with connective and interstitial tissue hyperplasia and empty seminiferous tubules. Spermatogenesis did not progress beyond spermatogonia (Figure 2B). It means that meiosis had not started at all. We observed no TUNEL-positive cells in the tubules of this male (Figure 3B). It means that meiosis had not started at all.

RK4 demonstrated an almost normal structure of the testes and content of the seminiferous tubules. Mature spermatozoa were present in the lumen of the tubules (Figure 2C, Supplementary Table 1). However, in some tubules of this male, we observed ball-like syncytial agglomerations of spermatids, while the spermatid-to-spermatocyte ratio was not disturbed (χ^2^ = 2.50, *p* = 1.00) (Figures 2D,E).

These aberrations were also present in five other hybrids: KR1, KR2, KR3, RK1, and RK3. The seminiferous epithelium cycle in these males shows a variety of disorders. In KR1 and KR3, the cycle was disrupted in some tubules. In RK1 and RK3, the cycle was disrupted in all tubules. Moreover, in KR1, RK1, and RK3, we detected a disorganized seminiferous tubule morphology.

The spermatid-to-spermatocyte I ratio was significantly reduced in the KR1, KR3, RK1, and RK3 hybrids (1.2 ± 1.3, χ^2^ = 148.88, *p* < 0.0001; 1.0 ± 0.9, χ^2^ = 147.50, *p* < 0.0001; 0.6 ± 0.6, χ^2^ = 45.80, *p* < 0.005; 0.4 ± 0.4, χ^2^ = 101.20, *p* < 0.0001, correspondingly). The spermatocyte I-to-spermatogonia ratio was higher than expected in KR1 (1.9 ± 1.0, χ^2^ = 37.03, *p* = 0.04) (Supplementary Table 1). These ratios indicate partial arrest of spermatogenesis before spermatid formation. Some seminiferous tubules were either empty or filled with cell debris. We detected spermatogenesis arrest at the meiotic prophase. It was partial in KR1, KR3, and RK4 and well expressed in RK1 and RK3. However, single abnormal mature spermatozoa were detected in the seminiferous epithelium of two of them: KR1 and RK3, while early spermatozoa were detected in the tubules of KR2, KR3, and RK1.

#### Hybrids Between *Microtus rossiaemeridionalis* and *Microtus mystacinus*

All the five hybrid males had the same set of aberrations: partial consolidation of interstitial tissue, disrupted seminiferous epithelium cycle progression in all tubules, disorganized seminiferous tubule morphology, and the absence of mature spermatids and spermatozoa (Figure 2F). In all of these hybrids, we detected an excess of primary spermatocytes followed by apoptosis (Figure 3C). The mean spermatocyte I-to-spermatogonia ratio was close to zero to one vs. four to one expected (χ^2^ = 264.44, *p* < 0.0001) (Supplementary Table 1). In male RM3, we observed erratic syncytial spermatid balls; in male RM2, we detected a seminoma in the germinal epithelium of the seminiferous tubules (Figure 2G).

#### Hybrids Between *Microtus arvalis* and *Microtus kermanensis*

We examined tree hybrids between *M. arvalis* “obscurus” and *M. kermanensis*. The hybrids showed variation in the degree of disturbances in spermatogenesis (Supplementary Table 1). AK2 and AK3 demonstrated tubule disorganization and a disrupted seminiferous epithelium cycle. Additionally, AK2 showed hyperplasia of interstitial tissue and apoptosis of primary spermatocytes. Some tubules had an excess of primary spermatocytes. In all tree hybrids, however, the mean spermatogonia-to-spermatocyte I ratio did not differ significantly from the expected 1:1 (1.9 ± 0.9, χ^2^ = 30.93, *p* = 1.0). AK1 had some abnormal spermatids and early spermatozoa in the seminiferous epithelium, while the other two hybrids lacked spermatids completely (Figure 2H).

#### Hybrids Between *Microtus kermanensis* and *Microtus transcaspicus*

*Microtus kermanensis* and *M. transcaspicus* were the most distant parental species in our experiment, yet their hybrids showed the same disturbances in spermatogenesis as the hybrids between the moderately distant *M. rossiaemeridionalis* and *M. mystacinus*. The testes of KT1 and KT2 showed normal morphology; however, the spermatogenic epithelium cycle was disputed. Most tubules displayed a disorganized and irregular structure of the germinal epithelium. Some tubules were empty or filled with cell debris and apoptotic primary spermatocytes. There was an excess of primary spermatocytes in all tubules (0.9 ± 0.9, χ^2^ = 213.77, *p* < 0.0001). Virtually no spermatids or spermatozoa were detected at the seminiferous epithelium of these hybrids (0.2 ± 0.6, χ^2^ = 427.22, *p* < 0.0001): not more than two early spermatids per field of view (Figure 2I and Supplementary Table 1).

#### Principal Component Analysis of Spermatogenesis in the Vole Hybrids

Based on the qualitative and quantitative characteristics of spermatogenesis, we observed the following situations with this process:

– Spermatogenesis proceeds to mature abnormal spermatozoa (KR1, KR2 and RK4 hybrids);
– Spermatogenesis proceeds to spermatids (KR3, RK1, RK3, and AK1 hybrids);
– Spermatogenesis is arrested at the spermatocyte I stage (AK2, AK3, and all KT, MR, RM hybrids);
– Spermatogenesis is arrested at the spermatogonia stage; seminiferous tubules are completely empty (RK2 hybrid).

The plot in Figure 2J represents the results of a PCA of histological data. RK2 had the most severe abnormalities (on the left side of the plot), while KR2 and RK4 were the least affected (at the right side of the plot). At the right half of the plot, there are hybrids with spermatogenesis proceeding to the spermatid or the spermatozoa stage, while in the bottom-left quarter, there is one big cluster of hybrids with a complete meiotic block at the leptotene–zygotene–pachytene stages. Thus, PC1, which explains 57.5% of the variance of the total variation, is in good agreement with the timing of spermatogenesis progression.

### Cytological Mechanisms of Hybrid Male Sterility

Analysis of the dynamics of spermatogenesis in the sterile interspecies hybrids revealed wide variety in the onset of spermatogenesis arrest between and within the crosses. To gain an insight into the cytological causes of hybrid sterility, we examined chromosome synapsis, recombination, and epigenetic chromatin modifications in the hybrids whose parental species differed in the degree of genetic and chromosomal divergence. We used immunolocalization of several proteins specifically expressed at different substages of the meiotic prophase. SYCP3 and SYCP1 label the axial/lateral and central elements of SC, correspondingly (Zickler, 2006), RAD51 marks the ends of 3′ single-stranded DNA overhangs, which resulted from DNA double-strand breaks (Neale and Keeney, 2006), MLH1 marks mature recombination nodules (Anderson et al., 1999), and γH2A.X marks unrepaired DNA double-strand breaks and induces meiosis-specific inactivation of unpaired chromatin (Schoenmakers et al., 2008; Turner, 2015).

**FIGURE 4:**
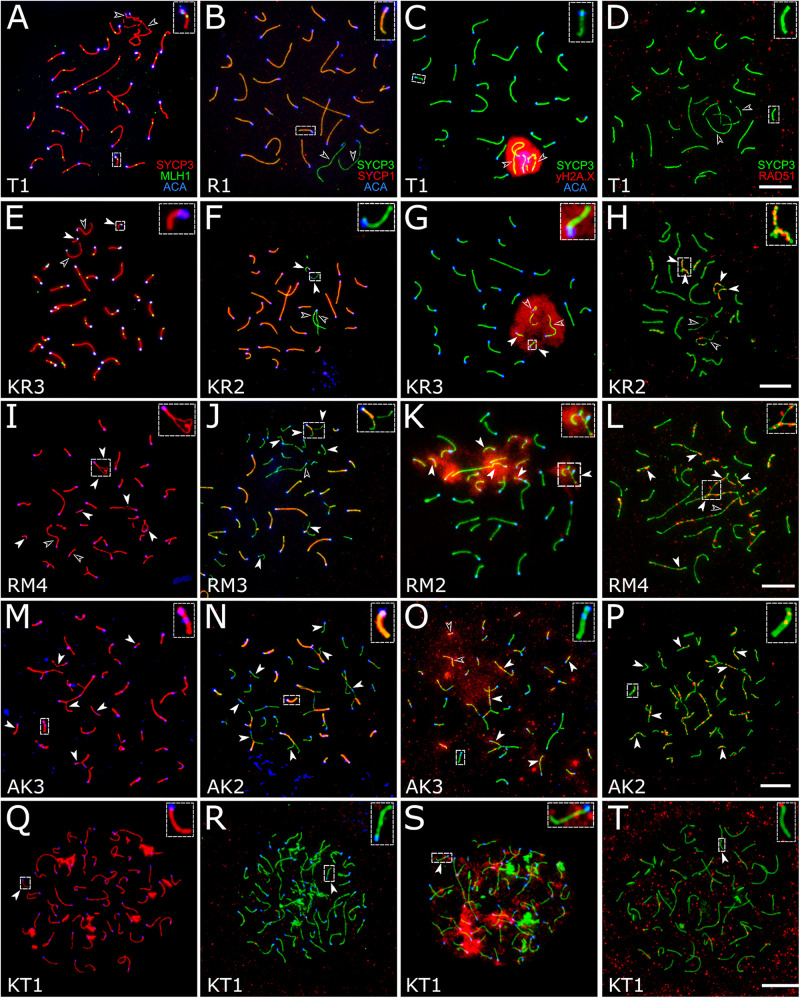
Spermatocytes of interspecies hybrid voles at different stages of prophase I. First row: the pachytene stage in the parental species [*M. transcaspicus* **(A–D)**]. Second row: the pachytene-like stage in the hybrids between species with the same karyotypes [*M. kermanensis* × *M. rossiaemeridionalis*, **(E–H)**]. Third row: the zygotene-like stage in the hybrids between species with the same karyotypes [*M. rossiaemeridionalis* × *M. kermanensis*, **(I–L)**]. Fourth row: the zygotene-like stage in the hybrids between species with different karyotypes [*M. arvalis* × *M. kermanensis*, **(M–P)**]. Fifth row: the leptotene-like stage in the hybrids between species with different karyotypes [*M. kermanensis* × *M. transcaspicus*, **(Q–T)**]. Columns show immunolocalization of the meiotic proteins: MLH1, SYCP1, γH2A.X, and RAD51, respectively. Arrowheads point to univalents or asynapsed chromosome regions; arrowhead outlines show distinguishable sex chromosomes. Letters and numbers in the bottom left corner of each image represent the type of cross and individual specimen number. Bar: 10 μm.

In the specimens from the parental species *M. transcaspicus*, *M. mystacinus*, *M. rossiaemeridionalis*, and *M. arvalis* “arvalis,” we observed orderly autosome synapsis and recombination, typical of the gray voles (Supplementary Tables 1, 7–9) (Borodin et al., 2012; Basheva et al., 2014; Torgasheva and Borodin, 2016). No autosomal univalents or multivalents were detected. The X and Y chromosomes were co-localized and formed a sex vesicle but did not synapse with each other. The sex vesicle was heavily labeled by γH2A.X antibodies, indicating its silencing (Figures 4A–D). This, however, should not be considered an aberration. X–Y asynapsis is a normal situation observed in all purebred species of the subgenus *Microtus* (Borodin et al., 2012).

#### Hybrids Between *Microtus kermanensis* and *Microtus rossiaemeridionalis*

There was a strong correspondence between the degree of histological and cytogenetic disturbances in spermatogenesis detected in the hybrids between *M. kermanensis* and *M. rossiaemeridionalis*. We found no spermatocytes in RK2, which had severely distorted testes. Conversely, RK4, which had an almost normal histological structure of the testes, showed an almost normal synapsis and recombination of most autosomes, similar to those observed in the *M. rossiaemeridionalis* male (Supplementary Table 1).

A small number of synaptic aberrations were found in the other hybrids: KR1, KR2, KR3, RK1, and RK3. Most of their pachytene-like cells contained most autosomes completely synapsed, with the central element of SC fully developed. Some small bivalents demonstrated a delayed synapsis of their ends (Figures 4E–G). About 12.7% (61 out 482) of cells contained up to six autosomal univalents (Supplementary Table 1). They usually appeared as small acrocentric and small metacentric chromosome pairs. The lateral elements of the univalents lacked SYCP1 (Figure 4F).

The chromatin of the unpaired autosomes and sex chromosomes was heavily labeled with γH2A.X antibodies, indicating the presence of unrepaired DNA double-strand breaks and transcriptional silencing (Figure 4G). Additionally, the asynapsed regions contained multiple foci of RAD51, another marker of unrepaired DNA double-strand breaks. A small number of RAD51 foci were detected in some fully synapsed bivalents (Figure 4H).

The recombination rate estimated as the number of MLH1 foci per cell containing no univalents was not reduced substantially in the hybrid males compared to the rate estimated in the purebred *M. rossiaemeridionalis* male (27.1 ± 1.4 and 27.5 ± 1.0, correspondingly, *t* = 1.6, *p*-value = 0.11) (Supplementary Table 1). This indicates a high level of homology between the genomes of the parental species and confirms the conclusions about a similarity of the karyotypes of the parental species based on comparisons of C- and NOR-band patterns of their chromosomes (Mahmoudi et al., 2018).

Thus, despite complete sterility, all but one reciprocal hybrid between *M. kermanensis* and *M. rossiaemeridionalis* showed nearly normal patterns of recombination and a rather low incidence of partial or complete asynapsis of some autosomes.

#### Hybrids Between *Microtus rossiaemeridionalis* and *Microtus mystacinus*

There was some variation in the degree of meiotic progression across the hybrids. Most germ cells of males RM1, RM3, and MR1 did not progress beyond the zygotene-like stage (Figure 4I). Univalents lacking SYCP1 signals and labeled with γH2A.X and RAD51 were the most common meiotic aberrations (Figures 4J–L). The fraction of completely unpaired autosomes varied across the hybrids from 13.8 to 66.2% (Figure 4I and Supplementary Table 1). Only 2.2% (four out of 179) of their cells contained all autosomal bivalents completely synapsed. Male RM4 showed a more advanced stage of meiotic prophase I. Eight percent (six out of 74) of its cells had recombination nodules marked by MLH1 antibodies, indicating that they had reached the mid-pachytene-like stage. The number of foci was substantially reduced (22.2 ± 2.9), because several chromosomes were present as univalents (Supplementary Table 1).

Despite having seminoma, RM2 male demonstrated an almost normal pattern of chromosome pairing similar to that described above for RK4, a hybrid between *M. kermanensis* and *M. rossiaemeridionalis*. The number of MLH1 foci observed in the pachytene cells of RM2 male was also similar to that detected in the reciprocal hybrids between *M. kermanensis* and *M. rossiaemeridionalis* (Supplementary Table 1).

#### Hybrids Between *Microtus arvalis* and *Microtus kermanensis*

These three hybrids show several signs of pairing failure. Chromosome pairing in these hybrids was complicated due to a substantial chromosomal divergence between the parental species (Figure 4M). High-resolution GTG-band karyotypes have been described in *M. arvalis* “obscurus” and *M. rossiaemeridionalis* (Mazurok et al., 2001), but not in *M. kermanensis*. What makes *M. arvalis* “obscurus” and *M. rossiaemeridionalis* different are one Robertsonian translocation, three tandem fusions, five pericentric inversions, and five centromeric shifts (Mazurok et al., 2001). Based on these data and on the assumption of karyotypic identity between *M. rossiaemeridionalis* and *M. kermanensis*, we expected to detect the following synaptic configurations in the *M. arvalis* × *M. kermanensis* hybrids: four trivalents, seven heteromorphic bivalents with one centromere at an end and another in the middle, one heteromorphic bivalent with two centromeres at the opposite ends, and 11 homomorphic bivalents (one metacentric and 10 acrocentric).

Among 146 cells examined, we observed none containing all these configurations at once. The spermatocyte best matching the expectations contained 11 homomorphic bivalents, eight partially synapsed heteromorphic bivalents, two trivalents, and six univalents. There were only two cells each containing all expected trivalents at once. In 6.1% of cells, we observed unexpected multivalents occurring due to non-homologous chromosome associations in pericentromeric regions. In one third of cells, no homomorphic bivalents were detected at all.

Univalents lacking SYCP1 signals were the most common meiotic aberration (Figure 4N). Their number varied from 5 to 48. Many cells contained odd numbers of univalents. This indicates that some of their partners were involved in non-homologous synapsis in multivalents. Thus, spermatocytes of *M. arvalis* × *M. kermanensis* hybrids varied in the number and types of synaptic aberrations and did not progress beyond the zygotene-like stage.

We detected minor occurrences of MLH1 foci in a few cells of these hybrids. The chromatin of univalents and asynapsed regions of bivalents and multivalents of all hybrids described in this section exhibited characteristic signs of unrepaired DNA double-strand breaks and transcriptional silencing: γH2A.X clouds (Figure 4O) and multiple foci of RAD51 antibodies (Figure 4P).

#### Hybrids Between *Microtus kermanensis* and *Microtus transcaspicus*

Spermatocytes of two male hybrids between the most genetically distant species *M. kermanensis* and *M. transcaspicus* had all signs of complete pairing failure consistent with our histological report (Figure 4Q). The cells contained only fragments of SC axial elements, indicating their incomplete assembly. In the most advanced spermatocytes, we observed almost normal axial elements, but all of them lacked SYCP1 signals and were not synapsed to each other (Figure 4R). γH2A.X clouds were abundant (Figure 4S). However, RAD51 signals were rather rare (Figure 4T). All these observations indicate that meiosis in the male hybrids between *M. kermanensis* and *M. transcaspicus* did not progress beyond the leptotene-like stage.

#### Comparative Analysis of Meiotic Aberrations in the Hybrid Voles

Table 4 summarizes typical meiotic aberrations detected in the interspecies hybrid male gray voles. As can be seen, the degree of chromosome pairing disturbances is roughly in concordance with the divergence time between the parental species (Figure 1). The hybrids between the most closely related species show sporadic asynapsis of several small bivalents, while the hybrids between the most distant species demonstrate complete synaptic failure. The more chromosome regions remain unpaired, and the more chromatin areas undergo silencing, as extensive immunolabeling with γH2A.X antibodies indicates.

**TABLE 4:** Meiotic aberrations detected in the interspecies hybrid male gray voles.

Species crossed	Number of specimens	Number of cells	Synaptic aberrations	Meiotic silencing of unsynapsed chromatin	Meiosis continues to
*M. kermanensis* × *M. rossiaemeridionalis*	6	482	Rare delayed synapsis or asynapsis of some small homologs	Rare univalents labeled with γH2A.X	Diplotene
*M. rossiaemeridionalis* × *M. mystacinus*	4	230	Asynapsis of part of the bivalent set	Multiple univalents and asynapsed chromosome regions labeled with γH2A.X	Zygotene
	1	17	Rare asynapsis of some small homologs	Rare univalents labeled with γH2A.X	Diplotene
*M. arvalis* × *M. kermanensis*	3	146	Incomplete synapsis of homologous chromosomes, presence of univalents, heteromorphic bivalents and trivalents	Multiple univalents and asynapsed regions of bi- and multivalents labeled with γH2A.X	Zygotene
*M. kermanensis* × *M. transcaspicus*	2	46	No synapsis	Abundant clouds of γH2A.X	Leptotene

## Discussion

### Species in the “*mystacinus*” Group Had Already Evolved Male Hybrid Sterility

The results of our breeding tests suggest that there was no intrinsic pre-copulative isolation between any pair of the parental species—at least not in captivity. This is an expected result. Allopatric species develop pre-copulative isolation mechanisms (behavioral, ecological, or morphological) much latter than sympatric species (Coyne and Orr, 2004). All interspecies crosses produced F1 litters. The litter sizes of the interspecies progeny were within the limits of variation characteristic of the parental species, suggesting normal viability of the hybrid embryos.

All species examined showed intrinsic postzygotic isolation in the form of hybrid sterility. This sterility is more advanced between *M. kermanensis* × *M. arvalis* and *M. kermanensis* × *M. transcaspicus* hybrids, with both sexes being sterile. These species show a substantial genetic and karyotypic divergence. *M. kermanensis* has a 6.1% difference in *cytb* sequence from *M. arvalis* and 7.8% from *M. transcaspicus* (Mahmoudi et al., 2017; Golenishchev et al., 2019); additionally, *M. kermanensis* does not share at least 14 chromosome rearrangements with *M. arvalis* and at least eight with *M. transcaspicus* (Mazurok et al., 2001).

The male hybrids between the *M. mystacinus* × *M. kermanensis*, *M. mystacinus* × *M. rossiaemeridionalis* and *M. kermanensis* × *M. rossiaemeridionalis* crosses were sterile as well, while females remained fertile. These three species have the same chromosome number and morphology. *M. mystacinus* and *M. kermanensis* are virtually indistinguishable from each other in C-band patterns and NOR distribution and differ from *M. rossiaemeridionalis* only in the C-band pattern (Mahmoudi et al., 2018). *Cytb* analysis also indicates a phylogenetic proximity of *M. kermanensis* and *M. mystacinus.* Golenishchev et al. (2019) estimated the phylogenetic distances (p-distances) between the mitochondrial *cytb* DNA sequences of *M. kermanensis*, *M. mystacinus*, and *M. rossiaemeridionalis* as 4.0–4.4%. Yet, these species have already developed genetic incompatibility leading to male hybrid sterility. Therefore, these taxonomic forms of the “mystacinus” group should be considered valid species.

Our results indicate a concordance between genetic and chromosomal divergence, on the one hand, and cross-species incompatibility, on the other hand. They add a new dimension to the results of Allen et al. (2020), who demonstrated a correlation between *cytb* distances and hybrid sterility in mammals. They suggested that F1 male hybrids between species, in which *cytb* DNA sequences differ from each other by more than 7.2% of base pairs, are usually sterile. When the *cytb* distance is greater than 12%, the hybrids of both sexes are sterile.

Our data indicate that the species of the subgenus *Microtus* reach cross-incompatibility, which causes uni- and bisexual hybrid sterility, at smaller genetic distances. Within the framework of this study, we cannot assess separate effects of karyotypic and genetic divergence, because they are parallel in our set of parental species. However, accumulation of different chromosomal rearrangements in different species of the gray voles apparently played a minor role in the genesis of hybrid sterility. For example, the chromosomal forms *M. arvalis* “arvalis” and “obscurus,” which differ in a series of pericentric inversions and centromere shifts (Mazurok et al., 1996, 2001; Basheva et al., 2014), produce fertile male and female hybrids, both in nature and in the laboratory (Meyer et al., 1996). Female hybrids between *M. arvalis* and *M. rossiaemeridionalis*, which differ from each other in at least for 14 chromosomal rearrangements, demonstrate almost normal chromosome pairing and recombination in some prophase oocytes (Torgasheva and Borodin, 2016) and can produce backcross progeny (although very rarely) (Gileva et al., 2001).

The breeding test indicated that female sterility occurs at a more advanced stage of genetic divergence than does male sterility. Earlier, Torgasheva and Borodin (2016) demonstrated that even when both sexes are sterile, male meiosis is affected more severely than female meiosis. They suggested that in the course of speciation, males proceed to sterility faster than females, but both sexes follow the same route.

### The Degree of Disturbances in Spermatogenesis in Hybrids Increases With an Increase in Genetic Distance Between the Parental Species

In our set of the hybrids, we estimated the degree of male hybrid sterility at different stages of genetic divergence *via* the number and spectrum of spermatogenic abnormalities. In general, the degree of disturbances in spermatogenesis in the hybrids increased with an increase in genetic distance between the parental species. On average, these aberrations were less severe in the hybrids between the species of “mystacinus” group than in the hybrids between the species of “mystacinus” group and the more distant species *M. arvalis* and *M. transcaspicus.*

In our reciprocal hybrids, we did not observe a pronounced asymmetry in hybrid sterility of different cross directions, which has been reported in mouse (Dzur-Gejdosova et al., 2012; Larson et al., 2016; Nishino et al., 2018) and equine interspecies hybrids (Chandley et al., 1975). However, within some groups of hybrids, we observed wide variety in the severity of disturbances in spermatogenesis. Histological analysis revealed homogenous groups of hybrids (Figure 2J) represented by the reciprocal hybrids of *M.* r*ossiaemeridionalis* × *M. mystacinus* and *M. kermanensis* × *M. transcaspicus*, and heterogeneous groups including the reciprocal hybrids of *M.* r*ossiaemeridionalis* × *M. kermanensis* and *M. arvalis* × *M. kermanensis*.

Hybrids between *M. rossiaemeridionalis* and *M. kermanensis* were the most variable group. It included one male with empty seminiferous tubules, one male with spermatogenesis proceeding to mature abnormal spermatozoa, and five males with intermediate degrees of spermatogenic abnormalities. This heterogeneity was unlikely to be due to a genetic polymorphism in the parental species for the genes to control sterility in the hybrids. First, we tried to minimize the genetic heterogeneity of the parental colonies by sampling a small number of founders from the same populations during the trapping time. Secondly, we observed a difference in sterility phenotypes between the sibs. A similar heterogeneity of hybrid sterility phenotypes was observed in the F1 hybrid males of dwarf hamsters. In some of them, spermatogenesis was arrested before the meiotic prophase, while in others, it proceeded until the formation of mature although abnormal spermatozoa (Ishishita et al., 2015; Bikchurina et al., 2018). Hybrid marsupials also demonstrated variation in the onset of spermatogenesis arrest, from spermatids to abnormal mature sperm (Close and Lowry, 1989).

The variable aberrations of seminiferous epithelium progression could be caused by dysregulation of genes responsible for the development of particular spermatogenesis stages (Davis et al., 2015). Many regulatory elements, transcriptional factors, and chromatin state regulators take part in the development of seminiferous epithelial cells. The interspecies divergence at these loci is likely to make spermatogenesis unstable and vulnerable to stochastic deviations in the testis microenvironment.

### Meiotic Arrest in the Hybrids Is Mainly Due to Chromosome Pairing Failure

We found that the severity of meiotic aberrations in the hybrids was consistent with the degree of disturbances in spermatogenesis. They both increased with an increase in the phylogenetic distances between the parental species. Most chromosomes of the hybrids between the closely related species *M. kermanensis* and *M. rossiaemeridionalis* showed normal synapsis and recombination. Only few smallest chromosomes demonstrated delayed synapsis or asynapsis. All chromosomes of the hybrids between the most distant species *M. kermanensis* and *M. transcaspicus* showed complete pairing failure.

Partial or complete asynapsis of homologous chromosomes is the most common meiotic aberration detected in mammalian hybrids (Chandley et al., 1974; Zong and Fan, 1989; Borodin et al., 1998; Bhattacharyya et al., 2013; Ishishita et al., 2015; Torgasheva and Borodin, 2016). This is probably due to a divergence of the genetic systems controlling generation and repair of DNA double-strand breaks caused by SPO11 nuclease at the beginning of meiotic prophase I.

The most advanced and detailed studies of this system have been carried out on fertile and sterile male hybrids between laboratory strains derived from two subspecies of the house mouse *Mus musculus musculus* and *M. musculus domesticus* (Mihola et al., 2009; Bhattacharyya et al., 2013; Davies et al., 2016; Smagulova et al., 2016; Mukaj et al., 2020). These studies demonstrated that the system controlling the position of double-strand breaks along the chromosomes involves tight interactions between the protein PRDM9, a chromatin remodeling factor, and its targets, recombination hotspot motifs (Baudat et al., 2010; Parvanov et al., 2010). PRDM9 and its targets undergo a fast-paced concerted evolution triggered by an erosion of recombination hotspots. As a result, the genetic structure and chromosomal localization of recombination hotspots changed rapidly in a genetically isolated population. The incompatibility of these systems in the hybrids results in an asymmetric distribution of double-strand breaks along homologous chromosomes in heterozygotes (Davies et al., 2016; Smagulova et al., 2016). This, in turn, affects RAD51-mediated single-strand invasion and homology search. Delayed repair of asymmetric breaks leads to disturbances in homologous synapsis and, ultimately, to pairing failure. Unrepaired DNA breaks trigger γH2A.X-mediated transcriptional inactivation of unpaired chromatin (Carofiglio et al., 2013). Depending on the amount of the unpaired chromatin, transcriptional inactivation may lead to immediate or delayed meiotic arrest (Turner et al., 2006). The fact that the humanized *Prdm9* allele could rescue synapsis and restore fertility confirms the *Prdm9*-dependent mechanism as a major one underlying hybrid sterility formation (Mukaj et al., 2020).

This series of unfortunate meiotic events might explain the difference in the severity of spermatogenic aberrations that we observed in our hybrids. In the hybrids between closely related species, we observed asynapsis or delayed synapsis of small chromosomes. This was apparently because of a proportionally smaller number of recombination hotspots located on these small chromosomes. If some of them were asymmetric, they failed to pair, while larger chromosomes with a higher number of hotspots had a higher probability of at least one symmetric break occurring per chromosome and initiating homologous synapsis. The phenomenon that smaller autosomes are more sensitive to synapsis failure was described in mice (Gregorova et al., 2018).

Although small unpaired chromosomes underwent transcriptional inactivation indicated by γH2A.X, the amount of silenced chromatin was low. It triggered neither immediate meiotic arrest nor apoptosis. As evidenced from histological analysis, spermatogenesis in some of these hybrids proceeded almost to term. However, even these minor synaptic disturbances lead to the production of abnormal and dysfunctional spermatozoa and, as breeding records indicate, to complete sterility. Turner et al. (2006) demonstrated that, in mice, an asynapsis of the sex chromosomes at pachytene results in substantial postmeiotic repression in spermatids. Bikchurina et al. (2018) also showed that X–Y asynapsis in the dwarf hamster hybrids was the main cause of their sterility.

An independent evolution of genetically isolated species leads to accumulation of incompatibilities between their systems controlling homology recognition, synapsis, and recombination. The proportion of asymmetric double-strand breaks decreases; the delay in their repair grows together with the amount of unpaired and transcriptionally inactivated chromatin. In the hybrids between the phylogenetically distant species, we observe massive chromatin silencing with γH2A.X and no synapsis initiation. As a result, meiosis in these hybrids is arrested at the earliest of meiotic prophase I and spermatogenesis does not proceed beyond early spermatocytes.

## Data Availability Statement

The original contributions presented in the study are included in the article/Supplementary Material, further inquiries can be directed to the corresponding author/s.

## Ethics Statement

The animal study was reviewed and approved by Ethics Committees on Animal Care and Use at Zoological Institute and the Institute of Cytology and Genetics.

## Author Contributions

TB collected and analyzed cytological data. FG trapped the animals, maintained the breeding colonies, and collected and analyzed breeding data. AM collected materials. EK and TB collected and analyzed histological data. TB, FG, and PB wrote the manuscript. FG and PB conceived and designed the study. All authors reviewed the manuscript.

## Conflict of Interest

The authors declare that the research was conducted in the absence of any commercial or financial relationships that could be construed as a potential conflict of interest.
