# Supplementary material for: Reproductive Isolation Between Taxonomically Controversial Forms of the Gray Voles (Microtus, Rodentia; Arvicolinae): Cytological Mechanisms and Taxonomical Implications
Source: Front Genet. 2021 May 10;12:653837. doi: 10.3389/fgene.2021.653837 (PMC8141921; doi:10.3389/fgene.2021.653837)
Supplement: Supplementary file 1 [file Data_Sheet_1.ZIP › Bikchurina_Supplementary_Materials/Bikchurina_Supplementary_Material_1.DOCX]

Supplementary Material 1

# Supplementary Data

Supplementary Data contain 260 words, 9 Supplementary Tables, 6 Supplementary Figures.

**Algorithm of qualitative traits description of the spermatogenesis for Principal Component Analysis**

Abbreviations used for cell types:

SER – Sertolli cells

SPG – spermotogonia

SPC – spermatocytes

SPTD – spermatids

SPZ – spermatozoa

KR – *Microtus kermanensis* (dams) x *M. rossiameridionalis* (sires)

RK – *M. rossiameridionalis* (dams) x *M. kermanensis* (sires)

RM – *M. rossiameridionalis* (dams) x *M. mystacinus* (sires)

MR – *M. mystacinus* (dams) x *M. rossiameridionalismanensis* (sires)

AK – *M. arvalis "obscurus"* (dams) x *M. kermanensis* (sires)

KT – *M. kermanensis* (dams) x *M. transcaspicus* (sires)

M – *M. mystacinus*

Algorithm is based on six qualitative traits describing gonadal morphology, seminiferous epithelium cycle, seminiferous tubule morphology and features of germ cell populations – SPC, SPTD and SPZ.

1. We made a universal description of given traits for all specimen examined.

2. Using block-schemas for each trait (Suppl. Fig. 1-6) we filled out the Suppl. Table 2.

At block-schemas traits is graded as:

- normal (N) - testis structure does not differ from that of a normal sexually active male vole during reproductive season;
- deviant (D) - the structure is different from normal, however remains recognizable;
- abnormal (A) - the structure of the testis is different from normal and hardly recognizable;
- empty (E) – a pathological form of seminiferous tubule organization, which leads to a distortion of testis structure or absence of specific cell type in the tubule.

3. We converted qualitative traits into rank criteria (Suppl. Table 3) where E = 0, A = 1, D = 2, N = 3.

4. We added into the table three ratios between the numbers of different cell types scored in each testis: SPG/SER, SPC/SPG and SPTD/SPC and used the converted data in PCA.

# Supplementary Figures and Tables

**Supplementary Table 1.** Summary reproductive, histological and cytogenetic data for each F1 specimen and their parental species. Table saved in .xlsx format.

**Supplementary Table 2.** Specimen description for each trait

| Group | Specimen | Gonadal morphology | Seminiferous epithelium cycle | Seminiferous tubule morphology | SPC | SPTD | SPZ |
| --- | --- | --- | --- | --- | --- | --- | --- |
| KR | KR1 | A | D | A | D | D | D |
| KR | KR2 | D | N | N | N | N | A |
| KR | KR3 | D | D | D | D | A | A |
| RK | RK1 | A | A | A | A | D | A |
| RK | RK2 | E | E | E | E | E | E |
| RK | RK3 | A | A | A | A | D | D |
| RK | RK4 | N | N | N | D | D | N |
| RM | RM1 | A | A | A | A | A | E |
| RM | RM2 | D | A | A | A | A | E |
| RM | RM3 | D | A | A | A | A | E |
| RM | RM4 | D | A | A | A | A | E |
| MR | MR1 | D | A | A | A | A | E |
| AK | AK1 | N | N | N | D | A | A |
| AK | AK2 | D | A | A | A | E | E |
| AK | AK3 | N | A | A | A | E | E |
| KT | KT1 | N | A | A | A | A | E |
| KT | KT2 | N | A | A | A | A | E |
| M | M1 | N | N | N | N | N | N |

**Supplementary Table 3.** Preparing data for PCA.

| Group | Specimen | Gonadal morphology | Seminiferous epithelium cycle | Seminiferous tubule morphology | SPC | SPTD | SPZ |
| --- | --- | --- | --- | --- | --- | --- | --- |
| KR | KR1 | 1 | 2 | 1 | 2 | 2 | 2 |
| KR | KR2 | 2 | 3 | 3 | 3 | 3 | 1 |
| KR | KR3 | 2 | 2 | 2 | 2 | 1 | 1 |
| RK | RK1 | 1 | 1 | 1 | 1 | 2 | 1 |
| RK | RK2 | 0 | 0 | 0 | 0 | 0 | 0 |
| RK | RK3 | 1 | 1 | 1 | 1 | 2 | 2 |
| RK | RK4 | 3 | 3 | 3 | 2 | 2 | 3 |
| RM | RM1 | 1 | 1 | 1 | 1 | 1 | 0 |
| RM | RM2 | 2 | 1 | 1 | 1 | 1 | 0 |
| RM | RM3 | 2 | 1 | 1 | 1 | 1 | 0 |
| RM | RM4 | 2 | 1 | 1 | 1 | 1 | 0 |
| MR | MR1 | 2 | 1 | 1 | 1 | 1 | 0 |
| AK | AK1 | 3 | 3 | 3 | 2 | 1 | 1 |
| AK | AK2 | 2 | 1 | 1 | 1 | 0 | 0 |
| AK | AK3 | 3 | 1 | 1 | 1 | 0 | 0 |
| KT | KT1 | 3 | 1 | 1 | 1 | 1 | 0 |
| KT | KT2 | 3 | 1 | 1 | 1 | 1 | 0 |
| M | M1 | 3 | 3 | 3 | 3 | 3 | 3 |

**Supplementary Table 4.** Importance of components.

| Component | Standard deviation | Eigenvalue variance | Proportion of Variance | Cumulative Proportion |
| --- | --- | --- | --- | --- |
| Dim.1 | 2.27 | 5.17 | 57.47 | 57.47 |
| Dim.2 | 1.27 | 1.61 | 17.91 | 75.38 |
| Dim.3 | 1.01 | 1.02 | 11.32 | 86.70 |
| Dim.4 | 0.71 | 0.51 | 5.66 | 92.36 |
| Dim.5 | 0.54 | 0.30 | 3.28 | 95.63 |
| Dim.6 | 0.49 | 0.24 | 2.65 | 98.29 |
| Dim.7 | 0.28 | 0.08 | 0.85 | 99.13 |
| Dim.8 | 0.26 | 0.07 | 0.74 | 99.88 |
| Dim.9 | 0.11 | 0.01 | 0.12 | 100.00 |

**Supplementary Table 5.** Traits contributions to the components.

| Trait | Dim1 | Dim2 | Dim3 | Dim4 | Dim5 | Dim6 | Dim7 | Dim8 | Dim9 |
| --- | --- | --- | --- | --- | --- | --- | --- | --- | --- |
| Gonad | 3.41 | 34.85 | 1.16 | 37.55 | 8.89 | 13.53 | 0.16 | 0.37 | 0.08 |
| Cycle | 17.59 | 1.95 | 0.05 | 0.03 | 3.31 | 15.08 | 4.69 | 4.28 | 53.03 |
| Tubule | 16.48 | 4.29 | 0.23 | 0.64 | 3.91 | 13.19 | 10.73 | 29.01 | 21.53 |
| SPC | 17.46 | 0.56 | 0.00 | 0.46 | 16.70 | 0.01 | 15.79 | 33.29 | 15.72 |
| SPTD | 13.31 | 9.08 | 0.86 | 1.96 | 3.59 | 50.46 | 20.29 | 0.14 | 0.32 |
| SPZ | 13.49 | 6.12 | 0.03 | 1.93 | 59.88 | 3.65 | 2.97 | 7.93 | 4.01 |
| SPG/SER | 0.82 | 42.48 | 0.00 | 52.00 | 1.04 | 1.63 | 1.79 | 0.22 | 0.03 |
| SPC/SPG | 0.09 | 0.31 | 96.52 | 0.79 | 0.08 | 0.46 | 1.63 | 0.07 | 0.03 |
| SPTD/SPC | 17.35 | 0.36 | 1.15 | 4.64 | 2.60 | 2.01 | 41.97 | 24.68 | 5.25 |

**Supplementary Table 6.** The frequency of different cell types in the testes of F1 vole hybrids and *M. mystacinus*. Table saved in .xlsx format.

**Supplementary Table 7.** The univalents, asynapsed and synapsed elements count separately for F1 hybrids between the species of the same karyotypes and their parental species. Table saved in .xlsx format.

**Supplementary Table 8.** The univalents, asynapsed and synapsed elements count separately for F1 hybrids between the species of different karyotypes. Table saved in .xlsx format.

**Supplementary Table 9.** The MLH1 foci count in the spermatocytes of F1 vole hybrids (MLH1 count per cell) and their parental species. Table saved in .xlsx format.

## Supplementary Figures


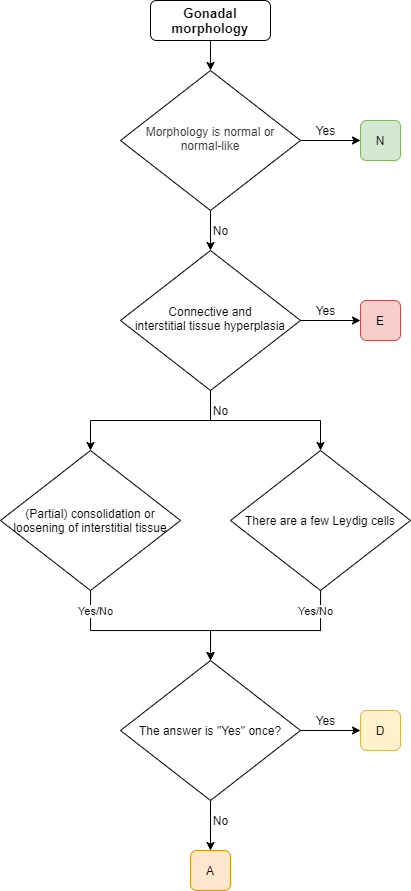


**Supplementary Figure 1.** Block-schema for Gonadal morphology trait.


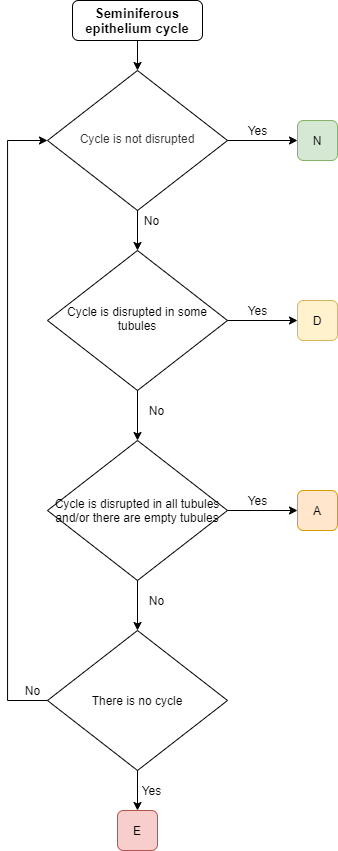


**Supplementary Figure 2.** Block-schema for Seminiferous epithelium cycle trait.


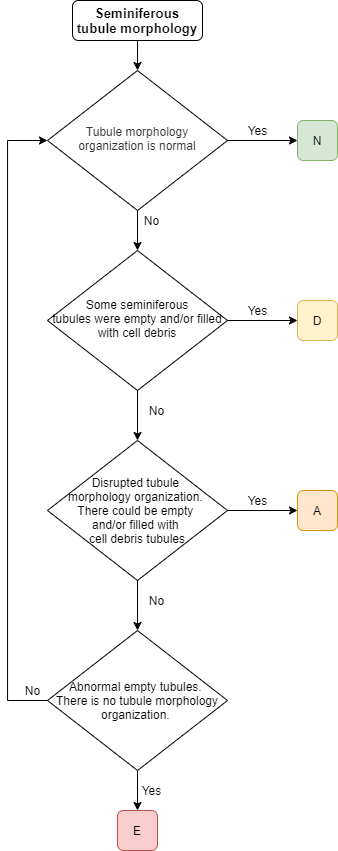


**Supplementary Figure 3.** Block-schema for Seminiferous tubule morphology trait.


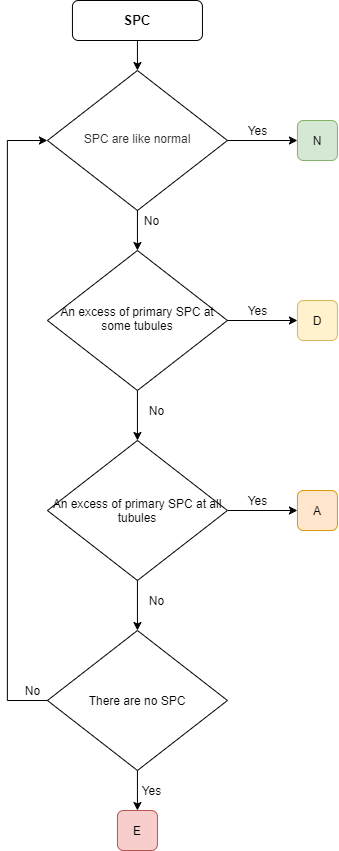


**Supplementary Figure 4.** Block-schema for SPC trait.


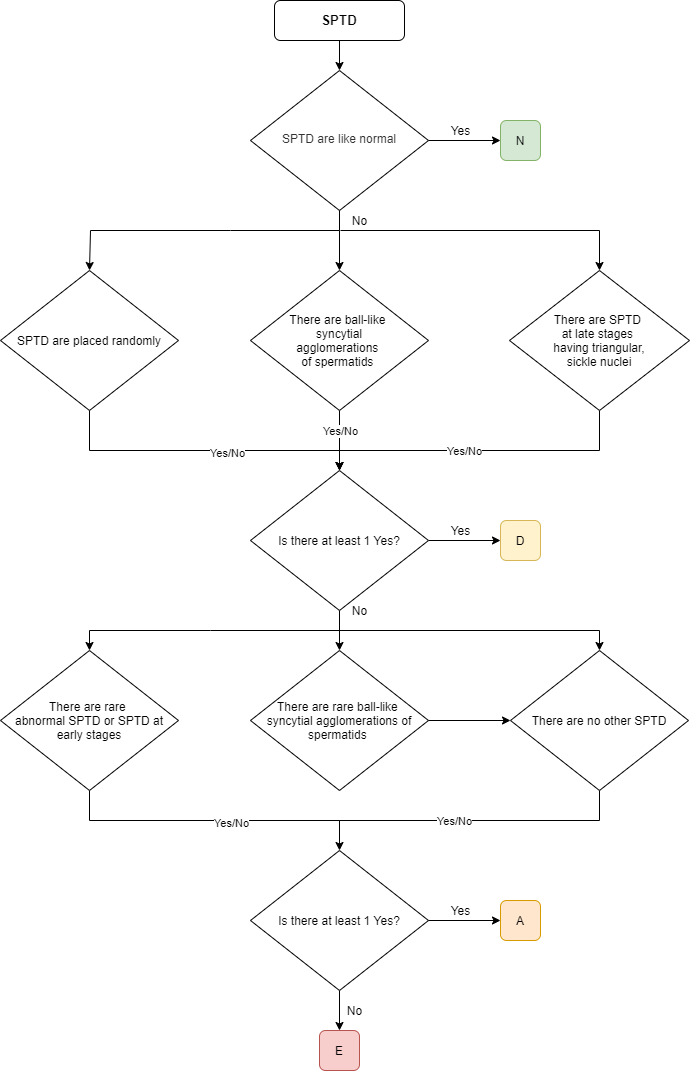


**Supplementary Figure 5.** Block-schema for SPTD trait.


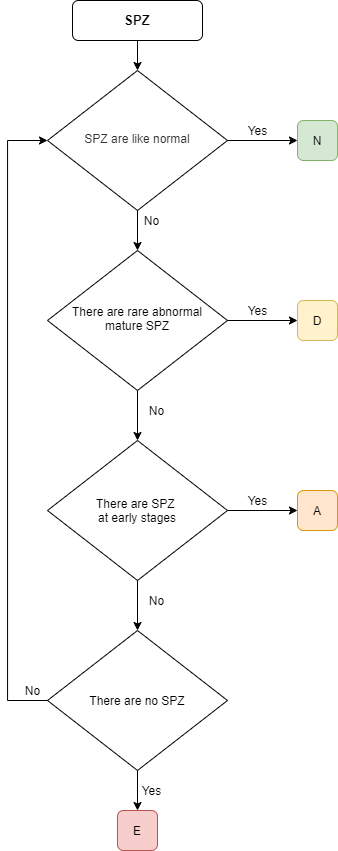


**Supplementary Figure 6.** Block-schema for SPZ trait.
