# Supplementary material for: Reproductive Isolation Between Taxonomically Controversial Forms of the Gray Voles (Microtus, Rodentia; Arvicolinae): Cytological Mechanisms and Taxonomical Implications
Source: Front Genet. 2021 May 10;12:653837. doi: 10.3389/fgene.2021.653837 (PMC8141921; doi:10.3389/fgene.2021.653837)
Supplement: Supplementary file 1 [file Data_Sheet_1.ZIP › Bikchurina_Supplementary_Materials/Bikchurina_Supplementary Material Presentation.zip!/Supplementary Material Presentation/Suppl_Figure_1.pdf]

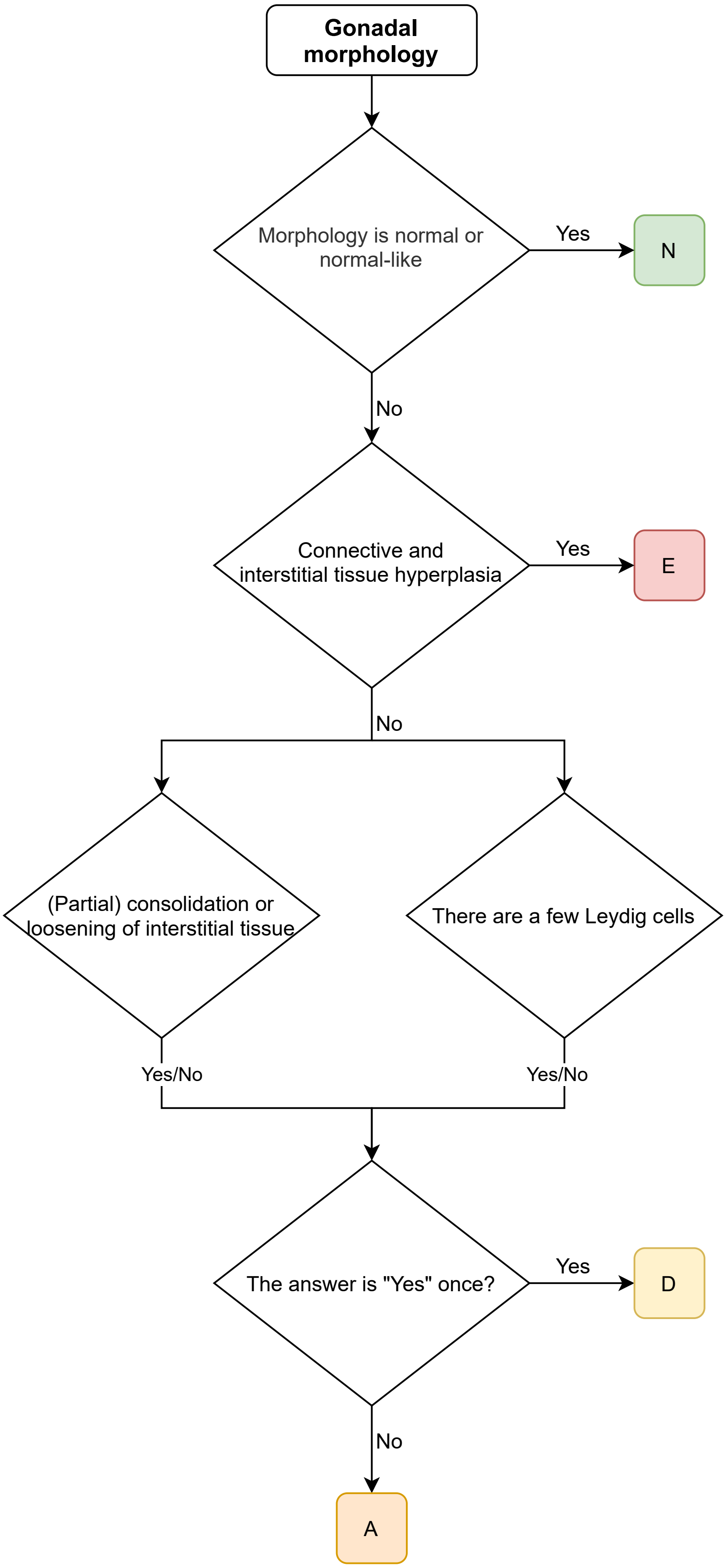
Gonadal morphology
Morphology is normal or normal-like
Yes
N
No
Connective and interstitial tissue hyperplasia
Yes
E
No
(Partial) consolidation or loosening of interstitial tissue
There are a few Leydig cells
Yes/No
Yes/No
The answer is "Yes" once?
Yes
D
No
A
