# Supplementary material for: Reproductive Isolation Between Taxonomically Controversial Forms of the Gray Voles (Microtus, Rodentia; Arvicolinae): Cytological Mechanisms and Taxonomical Implications
Source: Front Genet. 2021 May 10;12:653837. doi: 10.3389/fgene.2021.653837 (PMC8141921; doi:10.3389/fgene.2021.653837)
Supplement: Supplementary file 1 [file Data_Sheet_1.ZIP › Bikchurina_Supplementary_Materials/Bikchurina_Supplementary Material Presentation.zip!/Supplementary Material Presentation/Suppl_Figure_3.pdf]

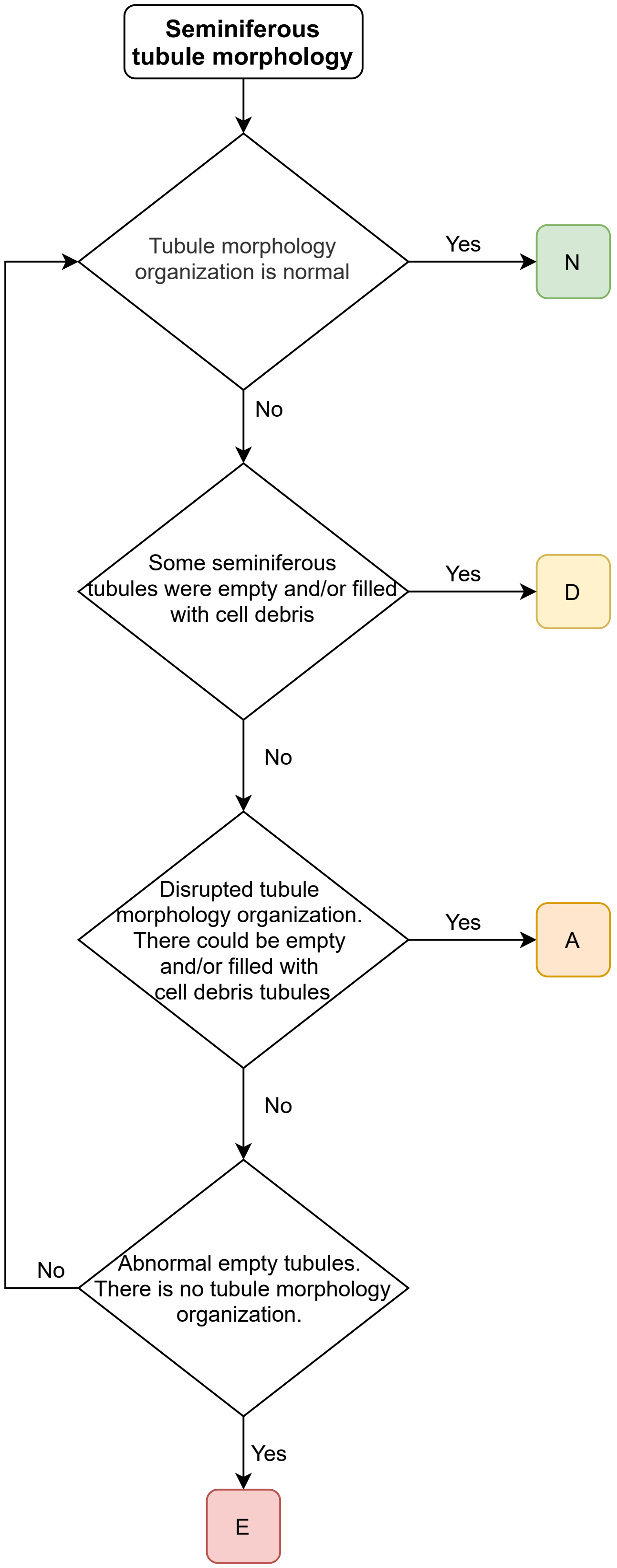
Seminiferous tubule morphology
Tubule morphology organization is normal
Yes
N
No
Some seminiferous tubules were empty and/or filled with cell debris
Yes
D
No
Disrupted tubule morphology organization. There could be empty and/or filled with cell debris tubules
Yes
A
No
Abnormal empty tubules. There is no tubule morphology organization.
No
Yes
E
