# Supplementary material for: Reproductive Isolation Between Taxonomically Controversial Forms of the Gray Voles (Microtus, Rodentia; Arvicolinae): Cytological Mechanisms and Taxonomical Implications
Source: Front Genet. 2021 May 10;12:653837. doi: 10.3389/fgene.2021.653837 (PMC8141921; doi:10.3389/fgene.2021.653837)
Supplement: Supplementary file 1 [file Data_Sheet_1.ZIP › Bikchurina_Supplementary_Materials/Bikchurina_Supplementary Material Presentation.zip!/Supplementary Material Presentation/Suppl_Figure_4.pdf]

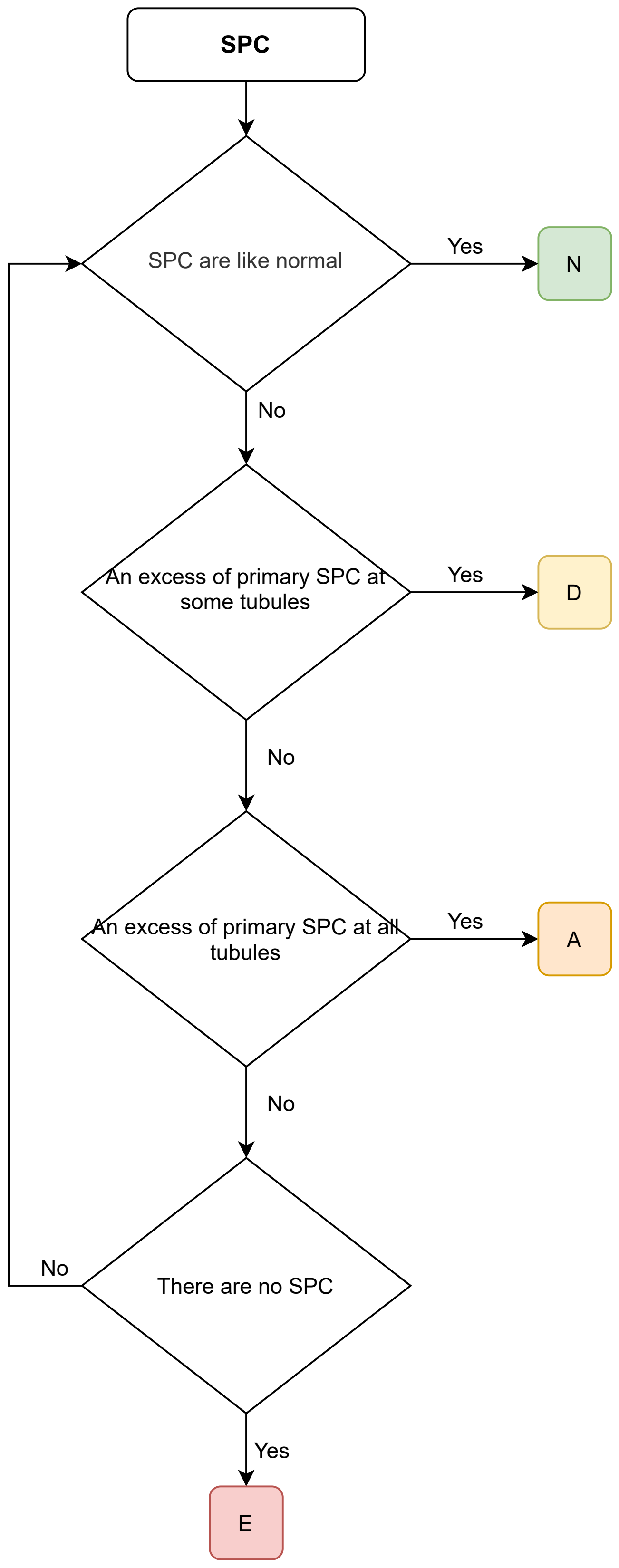
SPC
SPC are like normal
Yes
N
No
An excess of primary SPC at some tubules
Yes
D
No
An excess of primary SPC at all tubules
Yes
A
No
There are no SPC
No
Yes
E
