# Supplementary material for: Reproductive Isolation Between Taxonomically Controversial Forms of the Gray Voles (Microtus, Rodentia; Arvicolinae): Cytological Mechanisms and Taxonomical Implications
Source: Front Genet. 2021 May 10;12:653837. doi: 10.3389/fgene.2021.653837 (PMC8141921; doi:10.3389/fgene.2021.653837)
Supplement: Supplementary file 1 [file Data_Sheet_1.ZIP › Bikchurina_Supplementary_Materials/Bikchurina_Supplementary Material Presentation.zip!/Supplementary Material Presentation/Suppl_Figure_5.pdf]

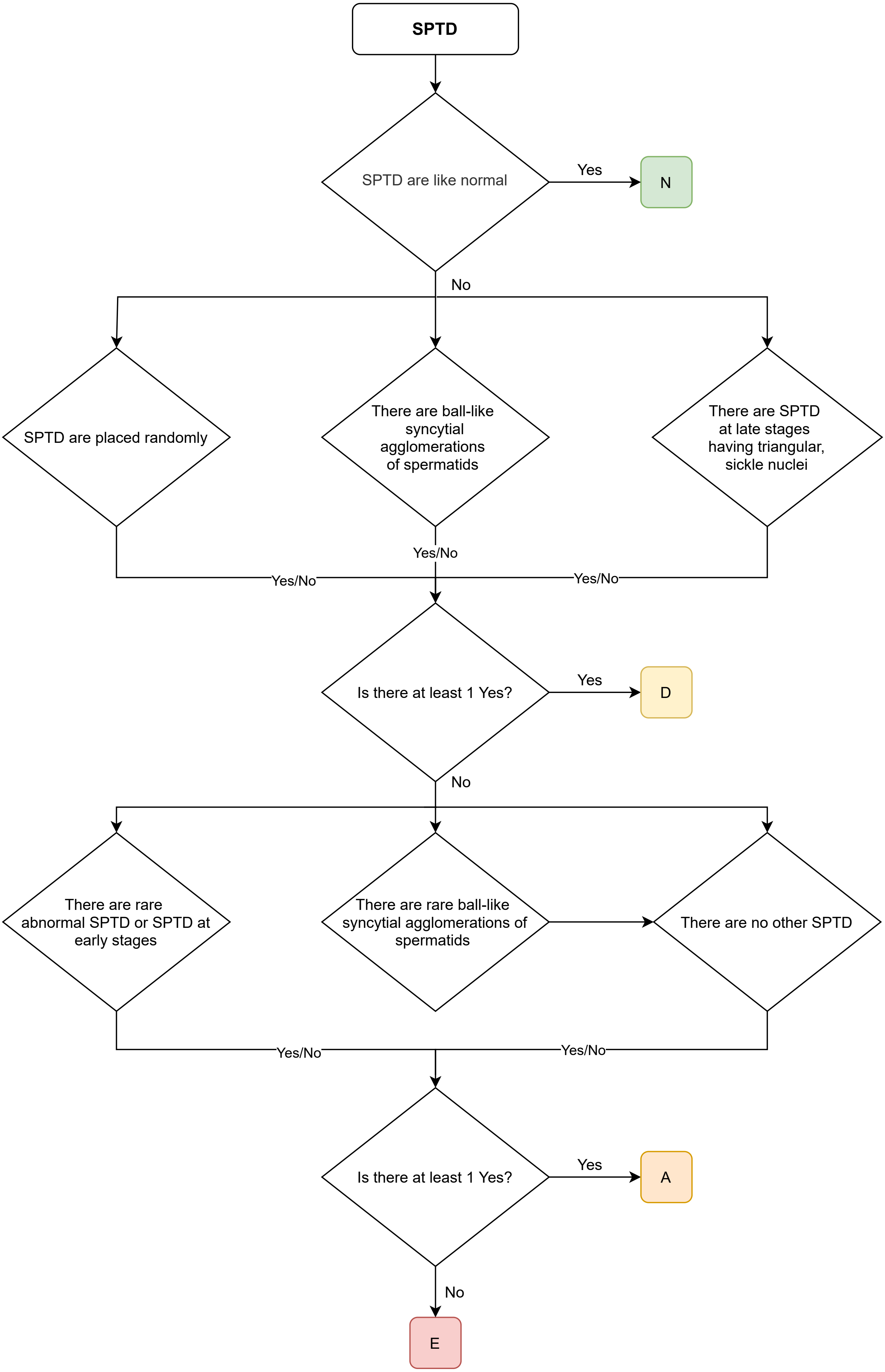
SPTD
SPTD are like normal
Yes
N
No
SPTD are placed randomly
There are ball-like syncytial agglomerations of spermatids
There are SPTD at late stages having triangular, sickle nuclei
Yes/No
Yes/No
Yes/No
Is there at least 1 Yes?
Yes
D
No
There are rare abnormal SPTD or SPTD at early stages
There are rare ball-like syncytial agglomerations of spermatids
There are no other SPTD
Yes/No
Yes/No
Is there at least 1 Yes?
Yes
A
No
E
