# Supplementary material for: Reproductive Isolation Between Taxonomically Controversial Forms of the Gray Voles (Microtus, Rodentia; Arvicolinae): Cytological Mechanisms and Taxonomical Implications
Source: Front Genet. 2021 May 10;12:653837. doi: 10.3389/fgene.2021.653837 (PMC8141921; doi:10.3389/fgene.2021.653837)
Supplement: Supplementary file 1 [file Data_Sheet_1.ZIP › Bikchurina_Supplementary_Materials/Bikchurina_Supplementary_Material_2.doc]

| **Specimen** | **Gonadal morphology** | **Seminiferous epithelium cycle** | **Seminiferous tubule morphology** | **Features of germ cell populations** | | |
| --- | --- | --- | --- | --- | --- | --- |
|  |  |  |  | **Spermatocytes (SPC)** | **Spermatids (SPTD)** | **Spermatozoa (SPZ)** |
| KR1 | Loosening of interstitial tissue. There are a few Leydig cells. | Cycle is disrupted in some tubules. | Disrupted tubule morphology organization. Tubules are empty and filled with cell debris. | There is an excess of primary SPC at some tubules. | SPTD are placed randomly. There are SPTD at late stages having triangular, sickle nuclei. | There are rare abnormal mature SPZ. |
| KR2 | Loosening of interstitial tissue. | Cycle is not disrupted. | Tubule morphology organization is normal. | SPC are like normal. | SPTD look like normal. | There are SPZ at early stages. |
| KR3 | Loosening of interstitial tissue. | Cycle is disrupted in some tubules. | Some seminiferous tubules are empty and filled with cell debris. | There is an excess of primary SPC at some tubules. | There are rare SPTD or SPTD at early stages. | There are SPZ at early stages. |
| RK1 | Loosening of interstitial tissue. There are a few Leydig cells. | Cycle is disrupted in all tubules. | Disrupted tubule morphology organization. Tubules are empty and filled with cell debris. | There is an excess of primary SPC at all tubules. | SPTD are placed randomly. There are ball-like syncytial agglomerations of spermatids. There are SPTD at late stages having triangular, sickle nuclei. | There are SPZ at early stages. |
| RK2 | Connective and interstitial tissue hyperplasia. | There is no cycle. | Abnormal empty tubules. There is no tubule morphology organization. | There are no SPC. | There are no SPTD. | There are no SPZ. |
| RK3 | Loosening of interstitial tissue. There are a few Leydig cells. | Cycle is disrupted in all tubules. | Disrupted tubule morphology organization. Tubules are empty and filled with cell debris. | There is an excess of primary SPC at all tubules. | SPTD are placed randomly. There are ball-like syncytial agglomerations of spermatids. There are SPTD at late stages having triangular, sickle nuclei. | There are rare abnormal mature SPZ. |
| RK4 | Morphology is normal-like. | Cycle is not disrupted. | Tubule morphology organization is normal. | There is an excess of primary SPC at some tubules. | SPTD are like normal. There are ball-like syncytial agglomerations of spermatids. | SPZ are like normal. |
| RM1 | Consolidation of interstitial tissue. There are a few Leydig cells. | Cycle is disrupted in all tubules and there are empty tubules. | Tubule morphology organization is disrupted. | There is an excess of primary SPC at all tubules. | There are rare SPTD or SPTD at early stages. | There are no SPZ. |
| RM2 | Partial consolidation of interstitial tissue. | Cycle is disrupted in all tubules. | Tubule morphology organization is disrupted. There are tubules full or partially filled with seminoma cells. | There is an excess of primary SPC at all tubules. | There are rare SPTD or SPTD at early stages. | There are no SPZ. |
| RM3 | Partial consolidation of interstitial tissue. | Cycle is disrupted in all tubules. | Tubule morphology organization is disrupted. | There is an excess of primary SPC at all tubules. | There are no SPTD. There are rare ball-like syncytial agglomerations of spermatids. | There are no SPZ. |
| RM4 | Partial consolidation of interstitial tissue. | Cycle is disrupted in all tubules. | Tubule morphology organization is disrupted. | There is an excess of primary SPC at all tubules. | There are rare SPTD or SPTD at early stages. | There are no SPZ. |
| MR1 | Consolidation of interstitial tissue. | Cycle is disrupted in all tubules. | Tubule morphology organization is disrupted. | There is an excess of primary SPC at all tubules. | There are rare SPTD or SPTD at early stages. | There are no SPZ. |
| M1 | Morphology is normal. | Cycle is not disrupted. | Tubule morphology organization is normal. | SPC are normal. | SPTD are normal. | SPZ are normal. |
| AK1 | Morphology is normal-like. | Cycle is not disrupted. | Tubule morphology organization is normal. | There is an excess of primary SPC at some tubules. | There are rare SPTD or SPTD at early stages. | There are SPZ at early stages. |
| AK2 | Loosening of interstitial tissue. | Cycle is disrupted in all tubules. | Tubule morphology organization is disrupted. | There is an excess of primary SPC at all tubules. | There are no SPTD. | There are no SPZ. |
| AK3 | Morphology is normal-like. | Cycle is disrupted in all tubules. | Disrupted tubule morphology organization. Tubules are empty and filled with cell debris. | There is an excess of primary SPC at all tubules. | There are no SPTD. | There are no SPZ. |
| KT1 | Morphology is normal-like. | Cycle is disrupted in all tubules. | Disrupted tubule morphology organization. Tubules are empty and filled with cell debris. | There is an excess of primary SPC at all tubules. | There are rare SPTD or SPTD at early stages. | There are no SPZ. |
| KT2 | Morphology is normal-like. | Cycle is disrupted in all tubules. | Disrupted tubule morphology organization. Tubules are empty and filled with cell debris. | There is an excess of primary SPC at all tubules. | There are rare SPTD or SPTD at early stages. | There are no SPZ. |
